# Accuracy and reliability of the AO/OTA classification for tibial shaft fractures

**DOI:** 10.1016/j.jcot.2024.102826

**Published:** 2024-11-19

**Authors:** Rasmus Stokholm, Peter Larsen, Jan Duedal Rölfing, Marie Arildsen, Christian Grundtvig Rasmussen, Rasmus Elsoe

**Affiliations:** a Department of Orthopedic Surgery, Aalborg University Hospital, Aalborg, Denmark; b Department of Occupational Therapy and Physiotherapy, Aalborg University Hospital, Aalborg, Denmark; c Department of Orthopedics, Aarhus University Hospital, Aarhus, Denmark

**Keywords:** Fracture, Classification, AO/OTA 42-, Tibial shaft

## Abstract

**Background:**

Available literature lacks information regarding the accuracy and reliability of the AO/OTA classification for tibial shaft fractures. This study aimed to assess the inter- and intra-observer agreement and accuracy of the AO/OTA 42 classification (4-signs) for adult patients with tibial shaft fractures.

**Materials and methods:**

The study design is an accuracy, inter- and intra-observer agreement study. Anterior posterior (AP) and lateral X-rays of the fracture were used in the examination. The raters comprised of two junior doctors and two orthopedic trauma consultants, who underwent patient scoring twice in a blinded and randomized set-up. A committee including two consultant orthopedic trauma surgeons, one consultant orthopedic radiologist, and one associate professor was established to represent the “gold standard.” The 3- and 4-signs AO/OTA 42 classification was used for classification.

**Results:**

A total of 101 patients were included. X-rays were available for all 101 patients. Based on the gold standard classification, AO/OTA 42-A1 (56 %) was the most common fracture type, followed by AO/OTA 42-A3 (14 %). The agreement at 4-signs, when comparing the four raters and the gold standard classification was between 75 % and 86 % (Choen's kappa 0.53 to 0.79). Choen's kappa coefficient at 4-sgns for intra-and inter-observer agreement was between 0.47 and 0.74 and 0.31 and 0.60, respectively.

**Conclusion:**

This study showed substantial to moderate accuracy of the 4-signs AO/OTA 42- classification for tibial shaft fractures. Intra-observation agreements at 4-signs showed moderate to substantial agreement with and without available CT scans. Inter-observer agreements at 4-signs showed moderate to substantial agreement with only X-rays available. Inter-observer agreements for CT scan at 4-signs showed slight to moderate agreements.

## 1 Introduction

In trauma orthopedics, several long-bone fracture registers have been established in recent decades to improve the overall quality of fracture treatment.^1^, ^2^, ^3^, ^4^, ^5^ Outcome measurements from fracture registers commonly include basic patient characteristics, treatment/surgical procedures, adverse events, mortality, and patient-reported outcomes.^1^, ^2^, ^3^, ^4^, ^5^ The classification of fractures provides an important framework for communication. It is essential to monitor epidemiology and quality of treatment.^6^^,^^7^ Consequently, a valid, reliable, and accurate fracture classification system is mandatory for a high-quality fracture register.

The International Classification of Diseases (ICD-10) is widely distributed as a classification system primarily intended for hospital administration.^8^ However, the ICD-10 classification does not capture important aspects of the different fracture types concerning treatment options and outcomes. For such, the Arbeitsgemeinshaft für Osteosynthesefragen/Orthopedic Trauma Association (AO/OTA) classification is commonly used to classify long-bone fractures.^9^^,^^10^ The AO/OTA classification is a alphanumeric classification system reporting on most bone fractures in a standardizes system.^9^^,^^10^

A common fracture of the long bones is the tibial shaft fracture, with an incidence of 16.9/100,000/year.^11^ At present, only a few studies have reported on the accuracy and reliability of the AO/OTA classification for tibial shaft fractures.^12^^,^^13^ The available literature on the AO/OTA classification for tibial shaft fractures lacks information on inter- and intra-observer reliability. It includes only smaller samples and information regarding the impact of computer tomography (CT) on the classification of the fractures is missing

This study aimed to assess the inter- and intra-observer agreement and accuracy of the AO/OTA 42 classification for adult patients with tibial shaft fractures.

The secondary and explorative aims were to investigate the value of adding a CT scan to the classification and determine the influence of doctors’ experience levels on classification.

## 2 Patients and methods

### 2.1 Study design

The study design is an accuracy, inter- and intra-observer agreement study for the AO/OTA 42- classification of tibial shaft fractures. The primary outcome measurement was considered AO/OTA 42- at four signs (A1-A3, B2-B3, and C2-C3), and the secondary outcome included the AO/OTA 42- at three signs (A, B, C).^10^ Throughout this manuscript: four signs was defined a reporting on all the four alphanumeric characters in the classification, as example 42-A1. Three signs were defined as reporting on the first 3 alphanumeric characters in the classification, as example 42-A.

The design and completion of the study were performed in compliance with the ten quality criteria for evaluating fracture classification systems outlined by Audigé in 2004.^14^

The study was approved by the National Ethics Committee (N- 2308163) and the Danish Data Protection Agency (ID 2023–093).

### 2.2 Participants

Consecutive patients admitted with tibial shaft fractures AO/OTA 42- (A1-A3, B2-B3, and C2-C3) between September 2020 and June 2023 were included. Patients from the northern region of Denmark served by Aalborg University Hospital (level 1, trauma center), and three regional hospitals (level 2, trauma center) (Hjoerring Hospital, Thisted Hospital, and Farsoe Hospital) were included. Patients under 18 years of age and pathological fractures were excluded.

A total of 101 patients were included. The mean age of the patients was 53.1, ranging from 18 to 96. The female gender represented 47 % (N = 47) of the study population.

### 2.3 The AO/OTA classification

The Arbeitsgemeinshaft für Osteosynthesefragen/Orthopedic Trauma Association (OA/OTA) classification compendium was developed in 1987 by Müller and updated last in 2018.^10^ The AO/OTA classification divides shaft fractures into three groups: A (simple), B (wedge), and C (multi-fragmented) (3-signs – defined as A, B, C)) and two/three subgroups 1,2 and 3 (4-signs – defined as A1-A3, B2-B3, C2-C3)) with complexity and severity increasing.^10^ The (most recent) 2018 version of the AO/OTA 42- classification was subject to the present study.^10^

### 2.4 Procedure

A total of four raters were used in the study. The raters comprised two junior doctors (both 4 years postgraduate) and two orthopedic trauma consultants representing a common clinical setting in the orthopedic departments. The orthopedic trauma consultants were familiar with the AO/OTA 42- classification from daily clinical practice, which was not true for the junior doctors. Both junior doctors attended the AO-basic and advanced courses prior to participating in the study. No training was given as part of the present study.

The three and four signs AO/OTA 42- classification for tibial shaft fractures were scored twice for each patient, including a washout period between the first and second examinations of minimum 18 days. The presenting order of patients was rearranged between the first and the second examination. The four raters were not allowed to discuss, comment, or talk during the examination of the X-rays. All raters were blinded to the clinical patient information.

Anterior posterior (AP) and lateral X-rays of the fracture were used in the examination. In cases where CT was available, raters were only allowed to examine the CT scan following the classification of the initial X-ray. The examinations based on X-rays and the examinations based on X-rays combined with CT scans were reported separately. 3D reconstructions were available for all CT scans.

A committee including two consultant orthopedic trauma surgeons, one consultant orthopedic radiologist, and one associate professor from the trauma department was established to represent the “gold standard.” The committee used the three and four sign AO/OTA 42- classification for tibial shaft fractures for each patient. The committee was allowed to use both X-rays and, if available, CT scans in the examination. Disagreements between the committee members were solved through discussion.

## 3 Statistics

### 3.1 Sample size

Based on kappa statistics from a previous study, a kappa value of 0.5 was expected.^13^ To achieve a 95 % confidence interval that did not span more than one category on the kappa coefficient scale presented by Landis and Koch, the width of the confidence interval was set to 0.2.^15^ Four raters were assumed and could be graded into seven ordered categories (4-signs). A sample justification was calculated, giving a sample size of 101.^16^

### 3.2 Statistics

Means and 95 % confidence intervals (CI) are given for the continuous data. Percentages are given for the categorical data. The inter- and intra-observer agreement was analyzed by Cohen's kappa. Kappa values were interpreted as >0.8 excellent, 0.79–0.60 substantial, 0.59–0.40 moderate, 0.39.0.20 slight, and <0.20 poor.^15^ The statistical analysis was performed with Stata (Version 17).

## 4 Results

X-rays were available for all 101 patients, and CT scans were available for 66 (65 %) patients.

Based on the gold standard classification, AO/OTA 42-A1 (56 %) was the most common fracture type, followed by AO/OTA 42-A3 (14 %) (Table 1).

**Table 1:** AO/OTA golden standard.

	1	2	3	Sum
42A	57 (56 %)	11 (11 %)	14 (14 %)	82 (81 %)
42B	*||*	6 (6 %)	2 (2 %)	8(8 %)
42C	*||*	4(4 %)	7 (7 %)	11 (11 %)

### 4.1 Accuracy

Based on standard X-rays, the percentage of agreement when comparing the four raters and the gold standard AO/OTA 42- classification was between 77 % and 89 % at three signs and 75 % and 86 % at four signs, respectively. Disagreement in accuracy was mostly observed between the AO/OTA groups B2 and C2 and between the B3 and C3 groups.

When adding a CT scan to the classification, the percentage of agreement when comparing the four raters and the gold standard AO/OTA 42- classification were between 76 % and 88 % at three signs and 57 % and 80 % at four signs, respectively. Results indicated that the use of CT scans in classification is not needed (Table 2).

**Table 2:** Accuracy (raters vs. golden standard).

Raters	AP and side X-rays (N = 101)				CT-scan and X-rays (N = 66)			
	3-signs		4-signs		3-signs		4-signs	
	Accuracy (%)	Kappa (95%CI)	Accuracy (%)	Kappa (95%CI)	Accuracy (%)	Kappa (95%CI)	Accuracy (%)	Kappa (95%CI)
1 (senior)	87	0.64 (0.45–0.79)	83	0,75 (0.65–0.85)	76	0.50 (0.36–0.64)	73	0.59 (0.45–0.73)
2 (senior)	89	0.70 (0.55–0.85)	86	0.79 (0.69–0.89)	88	0.70 (0.52–0.88)	80	0.69 (0.55–0.84)
3 (junior)	84	0.37 (0.14–0.61)	79	0.64 (0.51–0.77)	77	0.15(-0.08–0.39)	57	0.45 (0.27–0.64)
4 (junior)	77	0.22 (0.00–0.42)	75	0.53 (0.35–0.71)	76	0.23 (0.00–0.46)	71	0.42 (0.25–0.59)

Based on standard X-rays, Choen's kappa coefficient was between 0.22 and 0.70 at three signs and 0.53 and 0.79 at four signs, indicating substantial to moderate agreement (4-signs). In contrate to senior doctors who demonstrated moderate to substantial agreement junior doctors demonstrated slight agreement at three signs (Table 2).

Based on CT scans and standard X-rays, Choen's kappa coefficient was between 0.15 and 0.70 at 3- signs and between 0.42 and 0.69 at 4-signs, indicating substantial to moderate agreement (4-signs). In contrate to senior doctors who demonstrated moderate to substantial agreement junior doctors demonstrated slight to poor agreement at three signs (Table 2).

### 4.2 Inter- and intra-observer agreement

Based on standard X-rays, Choen's kappa coefficient for intra-observer agreement was between 0.50 and 0.61 at three signs and 0.57 and 0.74 at four signs, indicating substantial to moderate agreement between the two different ratings. When adding a CT scan to the classification, the Choen's kappa coefficient for the intra-observer agreement was between 0.48 and 0.65 at three signs and between 0.47 and 0.59 at four signs (Table 3).

**Table 3:** Inter- and intra-observer agreement.

Intra-observer	AP and side X-rays (N = 101)		CT-scan and X-rays (N = 66)	
	3-signs	4-signs	3-signs	4-signs
	Kappa (95%CI)	Kappa (95%CI)	Kappa (95%CI)	Kappa (95%CI)
R1 (senior)	0.51 (0.35–0.68)	0.61 (0.51–0.73)	0.50 (0.31–0.69)	0.47 (0.33–0.61)
R2 (senior)	0.50 (0.34–0.66)	0.61 (0.50–0.72)	0.49 (0.34–0.65)	0.50 (0.36–0.64)
R3 (junior)	0.61 (0.34–0.87)	0.74 (0.62–0.86)	0.48 (0.33–0.64)	0.59 (0.39–0.79)
R4 (junior)	0.58 (0.39–0.77)	0.57 (0.44–0.70)	0.65 (0.43–0.87)	0.58 (0.41–0.75)

Inter-observer	Seminar 1		Seminar 1	
	3-signs	4-signs	3-signs	4-signs
	Kappa (95%CI)	Kappa (95%CI)	Kappa (95%CI)	Kappa (95%CI)
R1vsR2	0.54 (0.40–0.68)	0.60 (0.48–0.70)	0.39 (0.26–0.52)	0.40 (0.27–0.53)
R1vsR3	0.22 (0.03–0.41)	0.46 (0.34–0.59)	0.25 (0.04–0.46)	0.40 (0.23–0.57)
R1vsR4	0.28 (0.08–0.47)	0.40 (0.29–0.78)	0.33 (0.10–0.56)	0.43 (0.27–0.59)
R2vsR3	0.24 (0.08–0.43)	0.50 (0.38–0.63)	0.20 (0.06–0.34)	0.31 (0.15–0.46)
R2vsR4	0.31 (0.11–0.51)	0.46 (0.34–0.58)	0.22 (0.02–0.42)	0.33 (0.19–0.47)
R3vsR4	0.55 (0.35–0.75)	0.57 (0.45–0.69)	0.50 (0.19–0.81)	0.53 (0.36–0.70)

Based on standard X-rays, the Choen's kappa coefficient for inter-observer agreement (seminar 1) was between 0.22 and 0.55 at three signs and between 0.40 and 0.60 at four signs, indicating substantial to moderate agreement between the four raters at four signs. Comparable results were observed when adding a CT scan to the classification (Table 3).

Due to non-overlapping confidence intervals, no significant difference in the inter-observer agreement (Cohen's kappa coefficient) was observed when comparing Seminar 1 to Seminar 2.

## 5 Discussion

Regardless of doctors’ previous experience level with the 4-signs AO/OTA 42- classification for tibial shaft fractures, this study showed that the accuracy was moderate to substantial. Adding a CT scan to the classification did not increase accuracy. Intra-observation agreements at 4-signs showed moderate to substantial agreement with and without available CT scans. Inter-observer agreements at 4-signs showed moderate to substantial agreement with only X-rays available. Inter-observer agreements for CT scan at 4-signs showed slight to moderate agreements.

### 5.1 Accuracy

This study showed high accuracy in using the AO/OTA 42- classification for adult tibial shaft fractures (>75 % at 4-signs). The Choen's kappa was between 0.49 and 0.79 at four signs, indicating moderate to substantial agreement. Disagreement in accuracy tends to be observed between the AO/OTA groups B2 and C2 and between the B3 and C3, indicating that fracture complexity may influence the accuracy of classification, which is commonly reported for other AO/OTA classifications.^13^^,^^17^ Moreover, this accuracy may be partly due to differences in the emphasis on either the fracture's comminution or the fracture's pattern.

The present study showed accuracy at comparable values compared to existing literature. Wennergren et al.^12^ included 32 patients and reported a Choen's kappa coefficient of 0.56 at four signs. Meling et al.^13^ included 26 patients and reported a Choen's kappa coefficient of 0.47 at four signs.

The Choen's kappa coefficient at three signs was observed with lower values compared to the four signs values, which is expected due to the number of AO/OTA groups (3 versus 7), as the Choen's kappa coefficient takes into account the possibility of the agreement occurring by chance.^18^

### 5.2 Inter- and intra-observer agreement

In the present study, the intra-observer agreement of the AO/OTA 42- classification for tibial shaft fractures was moderate to substantial (0.47–0.74). The inter-observer agreements showed light to substantial agreements with worse agreements when including junior doctors. Compared to existing literature, the present study showed intra- and inter-observer agreement at comparable or higher levels.^12^^,^^13^ However, to the author's knowledge, this study is the first to report the inter- and intra-observer agreement for the isolated AO/OTA 42- classification. Existing literature included scores for an agreement based on combined AO/OTA classifications (AO/OTA 41-, 42- and 43-), comparing results from the present study to existing literature with limited value.^12^^,^^13^

### 5.3 Doctors’ experience level

Regardless of classification based on standard X-rays or adding a CT scan, senior doctors tend to be more accurate compared to the gold standard classification. The 4-signs Choen's kappa coefficient based on standard X-rays were 0.53 and 0.64 for junior doctors and 0.75 and 0.79 for senior doctors. However, no statistically significant difference between doctors' experience levels was observed due to overlapping confidence intervals (Table 2). Results are supported by Melling et al.^13^ reporting that accuracy in the AO/OTA classification was not influenced by junior versus senior level experience. The influence of experience level in the AO/OTA classification is reported with conflicting results.^12^^,^^13^^,^^19^, ^20^, ^21^ Such difference may be explained by the differences in the familiarity of the AO/OTA classification regardless of experience level and by the reported difference in accuracy between different fracture types. Complex and shaft fractures are reported to be more difficult to classify than simple and end-segment fractures.^13^^,^^17^

### 5.4 Adding computer tomography (CT) to classification

This study is the first to report the significance of adding a CT scan to the AO/OTA 42- classification. In the present study, a preoperative CT scan was available in 66 of the 101 patients. Results showed comparable levels of accuracy between standard X-rays and CT scans added to standard X-rays in the classification. Comparable findings were observed for the inter- and intra-observer agreement.

Standard X-rays for the AO/OTA 42- classification of tibial shaft fractures are sufficient in clinical practice. Although existing literature included limited information regarding the significance of adding CT scans to increase accuracy in the AO/OTA classification, our results are in line with other research, reporting no significant improvement in the inter- and intra-observer agreement of the classification on other segments of the tibia, when utilizing CT scans (AO/OTA 41- and AO/OTA 43-).^19^^,^^22^ Moreover, adding a CT scan in the operative planning may be important in visualizing articular involvement.

### 5.5 Implications for clinical practice

A valid and reliable classification of tibial shaft fractures is essential for fracture registers and may also be useful in clinical practice. Tips and tricks in the surgical procedures treating tibial shaft fractures may differ between the different fracture types. Conveying these differences, using a valid classification system as a framework seems prudent. Consequently, a valid and reliable classification of tibial shaft fractures may also be useful in junior orthopedic doctors' planning and surgical training. Furthermore, a valid fracture classification may be useful when predicting outcome, as previous studies have indicated differences in outcomes between the different AO/OTA classifications^23^^,^^24^

### 5.6 Strengths and limitations

The major strengths of the present study are *i)*the large sample size based on a defined power calculation, ii)the inclusion of both X-rays and CT scans in the classification, and iii) the study was performed in compliance with the quality criteria proposed by Audigé et al.^14^ Compared to previous validity/reliability studies of the AO/OTA 42- classification of tibial shaft fractures, the present study included a much larger sample, and it is the first to report the importance of CT scan to the classification.^13^^,^^25^

However, some limitations should be mentioned. The true classification is not possible to validate. The gold standard was established by consensus between a committee of two consultant orthopedic trauma surgeons, one consultant orthopedic radiologist, and one associate professor from the trauma department. All members of the gold standard committee have several years of experience with the AO/OTA classification system. Furthermore, a large proportion of included fractures were classified as simple, which may have contributed to the moderate to substantial inter- and intra-observer agreement Another limitation may be the statistical power of selected AO/OTA subgroups at four signs, as some subgroups are only represented with a limited number of fractures. However, the confidence intervals are narrow compared to existing literature.

## 6 Conclusion

This study showed substantial to moderate accuracy of the AO/OTA 42- classification (4-signs) for tibial shaft fractures. Intra-observation agreements at 4-signs showed moderate to substantial agreement, with and without available CT scans. Inter-observer agreements at 4-signs showed moderate to substantial agreement with only X-rays available. Inter-observer agreements for CT scan at 4-signs showed slight to moderate agreements.

## Authors' contributions

Study design: RS, PL, RE, MA, CGR. Data collection: RS, PL, RE, MA, CGR. Data analysis, and wrote the manuscript: RS, PL, RE. Revision of manuscript and data interpretation: RS, PL, RE, MA, CGR. Approved the final version for publication: RS, PL, RE, MA, CGR.

## Funding

The project is partially funded by the Independent Research Fund Denmark. The role of the funding parties has been limited to granting financial support.

## Declaration of generative AI and AI-assisted technologies in the writing process’

Statement: No generative AI or AI-assisted technologies were used.

## Declaration of competing interest

The authors declare that they have no known competing financial interests or personal relationships that could have appeared to influence the work reported in this paper.
